# Editorial: Molecular and Cellular Pathways in NK Cell Development

**DOI:** 10.3389/fimmu.2020.01448

**Published:** 2020-07-14

**Authors:** Ewa Sitnicka, Yenan Bryceson, Aharon G. Freud, Emily M. Mace

**Affiliations:** ^1^Division of Molecular Hematology, Department of Laboratory Medicine, Lund University, Lund, Sweden; ^2^Department of Medicine, Center for Hematology and Regenerative Medicine, Karolinska Institutet, Stockholm, Sweden; ^3^Broegelmann Research Laboratory, Department of Clinical Sciences, University of Bergen, Bergen, Norway; ^4^Department of Pathology, The James Cancer Hospital and Solove Research Institute, The Ohio State University, Columbus, OH, United States; ^5^Comprehensive Cancer Center, The James Cancer Hospital and Solove Research Institute, The Ohio State University, Columbus, OH, United States; ^6^Department of Pediatrics, Columbia University Irving Medical Center, New York, NY, United States

**Keywords:** NK cells, developmental and maturation stages, regulatory pathways, disease-induced defects, clinical applications

We are delighted to present this Research Topic for *Frontiers in Immunology*, focusing on “Molecular and Cellular Pathways in NK Cell Development.”

This collection comprises five primary research articles, seven reviews of the current literature, and one opinion piece by experts in the field. Natural killer (NK) cells have immense therapeutic potential. Understanding how to acquire large numbers of functional cells and how to guide their activity is a focus of basic research with potential clinical application.

Papers included in this collection highlight recent advances in our understanding of NK cell origins, their cellular developmental stages and regulatory networks during normal hematopoiesis. These manuscripts also address molecular mechanisms responsible for NK cell defects found in patients with hematological malignancies and the degree to which NK cell impairments contribute to disease progression.

Despite having been discovered more than 40 years ago and used in the clinic for immunotherapy, several aspects of NK cell biology remain unexplored and are still being debated. In contrast to the mouse hematopoietic hierarchy, the development of human blood lineages is less characterized. Although the production and maintenance of NK cells are sustained by the pool of hematopoietic stem cells, the sites of NK cell development and the sequential intermediate differentiation stages are poorly defined. Cichocki et al. discuss two potential hierarchical models of human NK cell development: (1) a linear model where the lineage commitment occurs stepwise from hematopoietic stem cells, through the lymphoid–primed multilineage progenitors, the common-lymphoid progenitors, to NK cell-restricted progenitors and CD56^dim^ NK cells; and (2) a branched model where different NK cell populations, CD56^dim^, CD56^bright^, and adaptive NK cells, are generated from both early lymphoid and myeloid progenitors.

NK cells represent the founding member of a family of innate lymphoid cells (ILCs) and are placed within group 1. The ILC family consists of four subsets: NK cells/ILC1, ILC2, ILC3, and lymphoid tissue inducer cells. Stokic-Trtica et al. review the function, properties, diversity, and developmental relationship between NK cells and the other members of the ILC family. The authors summarize similarities and differences between NK cells and other ILCs, and discuss different potential therapeutic strategies to activate and harness anti-tumor immunity mediated by NK cells.

Among several transcription factors critical for NK cell development and maturation, Eomes represents a candidate that drives NK cell lineage-specification. O'Sullivan discusses heterogeneity within ILC1 cells in mice, where in addition to Eomes-dependent NK cells, there is a unique population of Eomes-independent ILC1s. This Eomes-independent ILC1 population represents a distinct lineage of group 1 ILCs rather than a developmental or functional stage of NK cells.

NK cells are heterogeneous in terms of their tissue location, phenotype, and function. In addition to the most abundant and the best studied conventional NK cells found in the blood and spleen, there are distinct subsets of tissue resident NK cells and helper ILC1s that have been identified in multiple organs and tissues including the liver, uterus, thymus, skin, and adipose tissue among others. Whereas, the development and regulation of bone marrow dependent conventional NK cells is well-characterized, the origin and regulation of recently described unique tissue-specific and tissue resident NK cells is less understood. Valero-Pacheco and Beaulieu provide a comprehensive overview of transcriptional regulatory pathways controlling and driving the development of tissue resident NK cells and helper ILC1s in mice.

To better characterize diverse populations of human NK cells, Filipovic et al. developed a 29-paremeter analysis panel to investigate NK cell subsets across three different tissues: liver, peripheral blood, and tonsil. This novel approach allows high dimensional profiling of NK cells in different tissues and can be applied as a potential diagnostic tool.

Adaptive NK cells represent a distinct long-lived population of NK cells that emerges after cytomegalovirus (CMV) infection providing the evidence for virus-specific NK cell immunological memory. Since NK cells are critical anti-viral effectors, these memory NK cells represent important potential therapeutic targets. In their studies, Gyurova et al. investigated changes in phenotype and function of NK cells from healthy individuals after treatment with CMV vaccine. Lack of changes in NKG2C^+^ NK cells was consistent with the absence of CMV infection, whereas the other NK cell subsets showed dynamic changes over time.

The origin and regulation of adaptive NK cells is not well-understood. Truitt et al. investigated CMV driven expansion of adaptive NK cells in rhesus macaques using lentivirally-barcoded autologous hematopoietic stem and progenitor transplantation that enabled tracking of CD56^−^CD16^+^ and CD56^+^CD16^−^ NK cell generation at the clonal level. The authors used this model to test the impact of infection on NK cell clonal dynamics and demonstrate long lasting clonal expansion in response to RhCMV, providing evidence for a clonal adaptive response and immunological memory within the NK cell compartment.

NK cell development and maturation have been driven and controlled by a network of cytokines (including: IL-2, IL-7, IL-12, IL-15, IL-21, IL-27, and interferons) that signal via the Janus kinase/signal transducer and activator of transcription (JAK/STAT) pathway. Gotthardt et al. review the current understanding of cytokine requirements and the downstream signaling involved in development and maturation of NK cells and ILC1s. The Authors also discuss the role of negative regulators of JAK/STAT signaling—the family of proteins called suppressor of cytokine signaling (SOCS) and their potential application as immunotherapeutic strategy. Scarno et al. previously applied next generation sequencing technology (NGS) to explore how JAK/STAT pathway regulate NK cells at different states of differentiation and function. The authors review how different STAT pathways are required in resting, effector and adaptive NK cells to control their expansion, differentiation, and function. Studies by Vian et al. further support the differential impact of cytokine signaling in NK cells and ICL1s, by demonstrating a high level of *Bcl2* expression in ILC1s after JAK inhibition compared to NK cells.

IL-15 role plays a central and unique role in NK cell biology. Pfefferle et al. review new insights into regulation of NK cell maturation and homeostasis, and discuss metabolic requirements, intra lineage NK cell plasticity, and transcriptional reprogramming of NK cells during differentiation and homeostatic proliferation in response to IL-15.

NK cells undergo phenotypic and functional changes in the presence of cytokines, and IL-2 has a crucial role in NK cell activation. Ranganath et al. have demonstrated that blocking IL-2 signaling by daclizumab beta used as a treatment for multiple sclerosis leads to the expansion of CD56^bright^ NK cells with enhanced ability to kill autoreactive T cells.

Ample data support a role of NK cells in tumor immune-surveillance and elimination of malignant transformed cells. There is clinical evidence supporting potent NK cell anti-tumor activity in the settings of chronic myeloid leukemia, acute myeloid leukemia, and myelodysplastic syndromes. However, disease-associated mechanisms induce NK cell defects and impairment in their cytotoxic function. Carlsten and Järås provide an overview of the mechanisms involved in disease-induced NK cell dysfunctions and discuss potential therapeutic approaches to restore NK cell function in patients with myeloid malignancies. They also discuss novel strategies to unleash NK cells against leukemic cells.

Together, the papers in this collection add new knowledge on the complex map of NK cell development, while also suggesting potential novel therapeutic strategies to modulate NK cell development and activity. These papers also lend new insights into how to endow NK cells with potent activity to control hematopoietic and non-hematopoietic malignancies.

We would like to take this opportunity to thank all the reviewers for their time and input. We also thank the authors for their valuable contributions to this Research Topic.

## Author Contributions

All authors listed have made a substantial, direct and intellectual contribution to the work, and approved it for publication.

## Conflict of Interest

The authors declare that the research was conducted in the absence of any commercial or financial relationships that could be construed as a potential conflict of interest.

